# The 6-month safety and efficacy of abatacept in patients with rheumatoid arthritis who underwent a washout after anti-tumour necrosis factor therapy or were directly switched to abatacept: the ARRIVE trial

**DOI:** 10.1136/ard.2008.099218

**Published:** 2008-12-14

**Authors:** M Schiff, C Pritchard, J E Huffstutter, V Rodriguez-Valverde, P Durez, X Zhou, T Li, K Bahrt, S Kelly, M Le Bars, M C Genovese

**Affiliations:** 1 University of Colorado, Denver, Colorado, USA; 2 Rheumatology Specialty Center, Willow Grove, Pennsylvania, USA; 3 Arthritis Associates, Hixson, Tennessee, USA; 4 Hospital Universitario Marques De Valdecilla, Universidad de Cantabria, Santander, Spain; 5 Cliniques Universitaires Saint-Luc, Université Catholique de Louvain, Brussels, Belgium; 6 Bristol-Myers Squibb, Princeton, New Jersey, USA; 7 Bristol-Myers Squibb, Rueil-Malmaison, France; 8 Stanford University, Palo Alto, California, USA

## Abstract

**Objective:**

To assess the safety, tolerability and efficacy of abatacept in patients with rheumatoid arthritis (RA) who had failed anti-tumour necrosis factor (TNF) therapy and were switched to abatacept directly or after completing washout.

**Methods:**

In this international, 6-month, open-label trial, patients had active RA, an inadequate response to anti-TNF therapy for 3 months or longer and a disease activity score in 28 joints (DAS28 (C-reactive protein; CRP) of 5.1 or greater. “Washout” patients discontinued anti-TNF therapy 2 months or longer pre-screening; “direct-switch” patients began abatacept (∼10 mg/kg) at their next scheduled anti-TNF therapy dose.

**Results:**

1046 patients were treated (449 washout, 597 direct-switch; baseline characteristics were similar between groups). At 6 months, adverse events (AE; 78.0% vs 79.2%), serious AE (11.1% vs 9.9%) and discontinuations due to AE (3.8% vs 4.0%) and serious AE (2.0% vs 1.3%) were comparable in washout versus direct-switch patients. There were no opportunistic infections. At 6 months, in washout versus direct-switch patients, similar clinically meaningful improvements were seen in DAS28 (CRP) (⩾1.2 unit improvement, 59.5% vs 53.6%, respectively; low disease activity state, 22.5% vs 22.3%; DAS28-defined remission, 12.0% vs 13.7%), physical function (health assessment questionnaire disability index ⩾0.22 improvement; 46.3% vs 47.1%) and health-related quality of life (mean change in short-form 36 scores: physical component summary, 5.5 vs 6.1; mental component summary, 4.8 vs 5.4).

**Conclusion:**

Abatacept demonstrated acceptable safety and tolerability and clinically meaningful efficacy over 6 months in patients with inadequate response to anti-TNF therapy. Results were comparable with or without a washout, supporting direct switching from anti-TNF therapy to abatacept as an option in clinical practice.

**Trial registration number:**

NCT00124982.

The efficacy and safety of abatacept, a selective T-cell co-stimulation modulator, has been demonstrated in patients with active rheumatoid arthritis (RA) and an inadequate response to methotrexate^1^ and/or anti-tumour necrosis factor (TNF) agents.^2^ In the Abatacept Trial in Treatment of Anti-TNF Inadequate Responders (ATTAIN) trial, patients with an inadequate response to anti-TNF agents were required to undergo a washout of their anti-TNF therapy before initiating abatacept. To date, no trial has evaluated abatacept treatment in patients who have switched directly from anti-TNF therapy without completing a washout period. This option may be more relevant in clinical practice.

The primary objective of the Abatacept Researched in RA patients with an Inadequate anti-TNF response to Validate Effectiveness (ARRIVE) trial was to assess the safety and tolerability of abatacept in patients with active RA who had failed up to three anti-TNF agents. Patients either completed a washout of their anti-TNF therapy or switched directly to abatacept. The ARRIVE trial included patients with RA who are representative of those typically encountered in clinical practice. Patients were eligible: (1) if they had failed anti-TNF therapy for safety or tolerability reasons alone; (2) if they had a positive purified protein derivative (PPD) test result (but had initiated treatment for latent tuberculosis and had a negative chest *x* ray); (3) irrespective of which background non-biological disease-modifying antirheumatic drug (DMARD) they were receiving; or (4) if they were receiving abatacept as monotherapy. (All patients who received abatacept as monotherapy were from the USA and were treated in accordance with the prescribing information for that country.) Here, we present the results from the first 6 months of the ARRIVE trial.

## Patients and methods

### Study population

Male and female patients with active RA^3^ ^4^ aged 18 years or older were enrolled in the USA, the European Union and Mexico. Patients were required to have had an inadequate response of at least 3 months to anti-TNF therapy, or to have discontinued anti-TNF therapy for safety or tolerability reasons. Patients were required to have a disease activity score in 28 joints (DAS28 (C-reactive protein; CRP)) of 5.1 or greater and were stratified into two groups according to anti-TNF therapy use before enrollment. “Washout” patients had discontinued anti-TNF therapy 2 months or more before screening, whereas “direct-switch” patients had received anti-TNF therapy within 2 months of screening, and received abatacept on their next scheduled anti-TNF therapy dose. Patients with an inadequate response to multiple anti-TNF therapies were included.

Patients were ineligible if they had evidence of or a recent history of disease associated with a major organ system, a serious infection or active tuberculosis requiring treatment within the past 3 years. Patients with a positive PPD test were eligible for the study if they had initiated treatment for latent tuberculosis one month or more before starting abatacept and had a negative chest *x* ray at enrollment.

### Study design

This was an international, phase IIIb, multicentre, open-label study in which all patients received a fixed dose of abatacept approximating 10 mg/kg on days 1, 15 and 29, and every 4 weeks thereafter up to and including day 141 (ClinicalTrials.gov identifier: NCT00124982).^5^ This was a 6-month trial with a long-term extension ending when the study medication was marketed in each country.

At study entry, patients were classified, via the central randomisation system, as “washout” if they had discontinued biological therapy for 2 months or more; otherwise, they were classified as “direct-switch”. Each centre in the study recruited patients for both treatment groups and enrollment was controlled so that both current and previous users were adequately represented.

Background non-biological DMARD were administered at the same dose and regimen at the time of randomisation; dose changes were not permitted unless for toxicity reasons. Before enrollment, all background non-biological DMARD therapies had to be stabilised for at least 28 days; oral corticosteroid treatment was stabilised for at least 25 out of 28 days. No biological DMARD therapies were allowed. Patients from the USA were permitted to receive abatacept as monotherapy.

The study began on 10 April 2005 and ended on 10 January 2007. The study was conducted in accordance with the Declaration of Helsinki and was approved by local institutional review boards. All patients provided written informed consent.

### Safety assessments

All patients who received at least one dose of study drug were evaluated for adverse events (AE), serious AE, clinically relevant changes in vital signs, laboratory test abnormalities and tolerability of abatacept.^6^

### Efficacy assessments

Disease activity (DAS28 (CRP))^7^ and physical function (health assessment questionnaire disability index; HAQ-DI)^8^ ^9^ were assessed on all visit days until day 169 or the early termination visit. A clinically meaningful improvement in disease activity was defined as a decrease from baseline of 1.2 or more units,^10^ a score 3.2 or less as low disease activity state (LDAS)^11^ and less than 2.6 as DAS28 (CRP)-defined remission.^12^ ^13^ A clinically meaningful response in physical function was defined as an improvement of 0.22 or more units.^14^ The short-form 36 (SF-36) measured health-related quality of life (HRQoL)^15^ with assessments made on visit days 1, 29, 85 and 169, or at the early termination visit. An improvement of 3 or more units was considered clinically meaningful.^15^

### Statistical analyses

The projected enrollment of 1000 treated patients allowed for the detection of at least one case of an uncommon AE at a frequency of 0.2%, with 86% probability. All available data from all patients who received at least one infusion of the study medication were included in the safety and efficacy datasets. Subgroup prespecified analyses were performed to evaluate the safety, tolerability and efficacy of abatacept in the washout versus direct-switch groups.

For safety analyses, frequencies of AE, serious AE and discontinuations due to AE/serious AE were summarised. For efficacy analyses, mean changes and 95% CI are presented for DAS28 (CRP), HAQ-DI and SF-36, based on patients with data available at the visit of interest (as-observed). For responder analyses (LDAS, DAS28-defined remission and HAQ-DI responders), data are based on all patients, with those who discontinued considered non-responders.

Post-hoc analyses were performed on the washout versus direct-switch groups to assess infections on a monthly basis for months 1, 2 and 3. Additional post-hoc analyses were performed on the overall population to assess efficacy by: (1) last anti-TNF agent used before the initiation of abatacept (etanercept, infliximab or adalimumab); (2) number of previous anti-TNF therapies (one, two or three); and (3) reason for anti-TNF therapy failure (safety, tolerability or efficacy). This study was not powered to detect differences in efficacy based on type, number or reason for failure of previous anti-TNF therapy; therefore, statistical testing was not performed.

## Results

### Baseline patient demographics and disease characteristics

**Figure 1:**
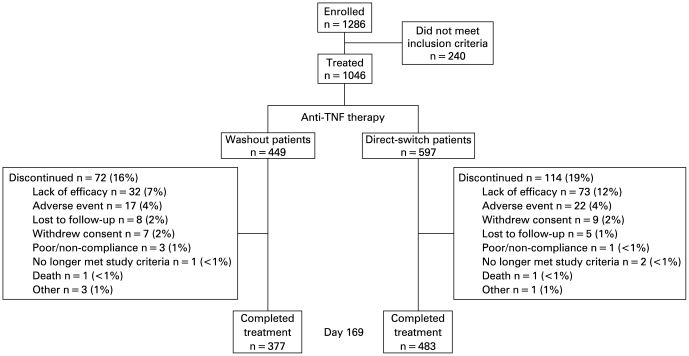
Patient disposition. TNF, tumour necrosis factor.

A total of 1286 patients were enrolled in the USA (103 sites), the European Union (32 sites) and Mexico (two sites). Of these, 1046 were treated (842 USA, 197 Europe, seven Mexico), with 449 in the washout and 597 in the direct-switch group (fig 1).

Baseline patient demographics and disease characteristics were similar for the washout and direct-switch groups (table 1). Of the patients in the direct-switch group who received infliximab before initiating abatacept, 16.3%, 29.7%, 28.5% and 25.6% had their last infliximab dose at less than 4, less than 6, less than 8 and approximately 8 weeks, respectively, before commencing abatacept. At enrollment, the mean number (SD) of anti-TNF therapies used by washout and direct-switch patients was 1.86 (0.97) and 1.95 (0.93), respectively. A total of 488 (46.7%), 340 (32.5%) and 200 (19.1%) patients had previously received one, two or three previous anti-TNF therapies (18 patients did not have available data). The most frequent background non-biological DMARD in use on study day 1 was methotrexate (table 1). Forty-three patients from the USA (20/449 (4.5%) washout and 23/597 (3.9%) direct-switch patients) were not receiving background non-biological DMARD therapy during the 6-month study period and thus received abatacept as monotherapy. (All patients who received abatacept as monotherapy were from the USA and were treated in accordance with the prescribing information for that country.)

**Table 1:** Baseline demographics and disease characteristics by washout and direct-switch groups

	Washout patients (n = 449)	Direct-switch patients (n = 597)	Overall (n = 1046)
Age, years	56.1 (12.5)	53.2 (12.3)	54.4 (12.4)
Weight, kg	78.9 (21.1)	79.1 (20.0)	79.0 (20.5)
Gender, female, n (%)	359 (80.0)	490 (82.1)	849 (81.2)
Race, n (%)			
White	416 (92.7)	551 (92.3)	967 (92.4)
Black	27 (6.0)	33 (5.5)	60 (5.7)
Other	6 (1.3)	13 (2.2)	19 (1.8)
Duration of disease, years	13.0 (10.0)	10.6 (9.0)	11.6 (9.5)
Previous anti-TNF therapy*, n (%)			
Etanercept	226 (50.3)	366 (61.3)	592 (56.6)
Infliximab	281 (62.6)	339 (56.8)	620 (59.3)
Adalimumab	193 (43.0)	309 (51.8)	502 (48.0)
Medications at day 1, n (%)			
Methotrexate	307 (68.4)	423 (70.9)	730 (69.8)
Azathioprine	18 (4.0)	25 (4.2)	43 (4.1)
Gold	4 (0.9)	1 (0.2)	5 (0.5)
Hydroxychloroquine/chloroquine	71 (15.8)	86 (14.4)	157 (15.0)
Leflunomide	66 (14.7)	68 (11.4)	134 (12.8)
Sulfasalazine	32 (7.1)	60 (10.1)	92 (8.8)
Corticosteroids†	280 (62.4)	331 (55.4)	611 (58.4)
Tender joints	17.8 (5.9)	17.8 (6.1)	17.8 (6.0)
Swollen joints	13.9 (5.6)	13.5 (5.4)	13.6 (5.5)
Patient global assessment, VAS 100 mm	72.7 (16.7)	73.1 (16.4)	72.9 (16.5)
HAQ-DI	1.7 (0.6)	1.7 (0.6)	1.7 (0.6)
DAS28 (CRP)‡	6.2 (0.7)	6.2 (0.7)	6.2 (0.7)
CRP (mg/dl)§	2.2 (3.0)	2.1 (3.0)	2.1 (3.0)
Rheumatoid factor positive, n (%)	292 (65.0)	349 (58.5)	641 (61.3)
PPD positive, n (%)	9 (2.0)	17 (2.8)	26 (2.5)

Data are presented as mean (SD) unless otherwise indicated. *Patients could have previously received more than one prior anti-tumour necrosis factor (TNF) therapy; two patients, one in the washout and one in the direct-switch group, had received rituxmab more than 12 months before study entry. †Oral and/or injectable. ‡The joint count used was 28. §The upper limit of normal for high sensitivity C-reactive protein (CRP) was 3.00 mg/l. DAS28, disease activity score in 28 joints; HAQ-DI, health assessment questionnaire disability index; PPD, purified protein derivative; VAS, visual analogue scale.

The mean (SD) duration of exposure to abatacept was similar for washout and direct-switch patients (6.2 (1.0) and 6.1 (1.1) months, respectively); the mean (SD) number of infusions was 6.4 (1.2) for both groups. Overall, 860 patients (82%) completed 6 months of treatment, with similar proportions among washout and direct-switch patients (fig 1). In the washout and direct-switch patients, respectively, the main reasons for discontinuation were lack of efficacy and AE (fig 1).

### Safety

A summary of safety to 6 months is presented in table 2. The most frequent AE (occurring in ⩾5% of patients in either group) were upper respiratory tract infection, headache, nausea, sinusitis, diarrhoea, bronchitis and fatigue. Serious AE occurred in 10.4% of patients overall, and in a similar proportion of patients in each group (11.1% and 9.9% of washout and direct-switch patients, respectively). The most frequent serious AE (excluding RA and osteoarthritis) were pneumonia (four direct-switch patients, 0.7%) and bronchitis (two washout patients, 0.4%; and one direct-switch patient, 0.2%), invertebral disc protrusion (three direct-switch patients, 0.5%) and myocardial infarction (one washout patient, 0.2%; and two direct-switch patients, 0.3%). There were two deaths: one caused by congestive heart failure (a 54-year-old female washout patient with a history of cardiomegaly and vascular congestion) and one caused by cardiac arrest (a 67-year-old male direct-switch patient with a history of hypertension).

**Table 2:** Safety summary at 6 months for washout and direct-switch patients

Patients with AE, n (%)	Washout patients (n = 449)	Direct-switch patients (n = 597)	Overall (N = 1046)
Total patients with AE	350 (78.0)	473 (79.2)	823 (78.7)
Total infections	176 (39.2)	231 (38.7)	407 (38.9)
Discontinuations due to AE	17 (3.8)	24 (4.0)	41 (3.9)
Serious AE	50 (11.1)	59 (9.9)	109 (10.4)
Total serious infections	12 (2.7)	13 (2.2)	25 (2.4)
Most frequent serious infections			
Pneumonia*	0	4 (0.7)	4 (0.4)
Bronchitis*	2 (0.4)	1 (0.2)	3 (0.3)
Lobar pneumonia*	2 (0.4)	0	2 (0.2)
Discontinuations due to serious AE	9 (2.0)	8 (1.3)	17 (1.6)
Autoimmune disorders	4 (0.9)	9 (1.5)	13 (1.2)
Total neoplasms†	8 (1.8)	7 (1.2)	15 (1.4)
Total malignancies	4 (0.9)	2 (0.3)	6 (0.6)
Basal cell carcinoma	0	2 (0.3)	2 (0.2)
Breast cancer	2 (0.4)	0	2 (0.2)
Lung adenocarcinoma	1 (0.2)	0	1 (0.1)
Uterine cancer	1 (0.2)	0	1 (0.1)

*Most frequent serious infections, reported in more than one patient in the overall population; all others occurred in one patient or fewer overall. †Benign, malignant and unspecified. AE, adverse event.

#### Infusional reactions

Acute infusional reactions (within one hour of the start of infusion) occurred in 20 (4.5%) washout patients and 37 (6.2%) direct-switch patients, the most frequent of which were dizziness (two washout and 10 direct-switch patients), headache (two washout and 10 direct-switch patients), hypertension (five washout and two direct-switch patients), nausea (one washout and eight direct-switch patients) and pruritis (two washout and three direct-switch patients). One acute infusional event was classified as severe (direct-switch group), presenting as a rash requiring treatment and leading to drug discontinuation.

#### Infections

Overall, infections were reported in 38.9% of patients (39.2% and 38.7% washout and direct-switch patients, respectively), the most frequent of which were upper respiratory tract infection (8.9% and 7.4% of washout and direct-switch patients, respectively), sinusitis (6.2% and 6.0% of washout and direct-switch patients, respectively) and bronchitis (5.6% and 3.7% of washout and direct-switch patients, respectively). The frequency of serious infections was similar between groups, at 2.7% and 2.2% among washout and direct-switch patients, respectively (table 2). No other serious infection occurred in more than one patient in the study. No cases of opportunistic infections, including tuberculosis, were reported in either group. Frequencies of infections by month were comparable for washout and direct-switch patients (11.1% vs 11.4% in month 1, 8.2% vs 10.4% in month 2 and 7.8% vs 6.4% in month 3, respectively). Overall, the frequencies of serious infections during months 1, 2 and 3 were 0.4%, 0.6% and 0.7%, respectively; for washout and direct-switch patients, frequencies were 0% versus 0.7% in month 1, 0.9% versus 0.3% in month 2 and 1.1% versus 0.3% in month 3, respectively.

#### Neoplasms

There were four (0.9%) neoplasms reported in the washout compared with two (0.3%) in the direct-switch group (table 2). There were four reports of non-skin malignancies (all in washout patients), including two patients with breast cancer, one with lung adenocarcinoma and one with uterine cancer (table 2).

#### Autoimmune disorders

The frequency of autoimmune disorders was slightly higher in the direct-switch compared with the washout group (nine patients (1.5%) vs four patients (0.9%), respectively). The most commonly occurring autoimmune disorder was psoriasis (one mild and one moderate case in washout patients; two mild cases in direct-switch patients). Three of these cases were new onset, whereas one patient had a history of psoriasis. All other types of autoimmune disorders were reported in no more than two patients across groups, were not severe and comprised: erythema nodosum, Sjogren’s syndrome, sicca syndrome, autoimmune thyroiditis (all two patients each) and keratoconjunctivitis sicca (one patient).

### Efficacy

#### Clinical efficacy

**Figure 2:**
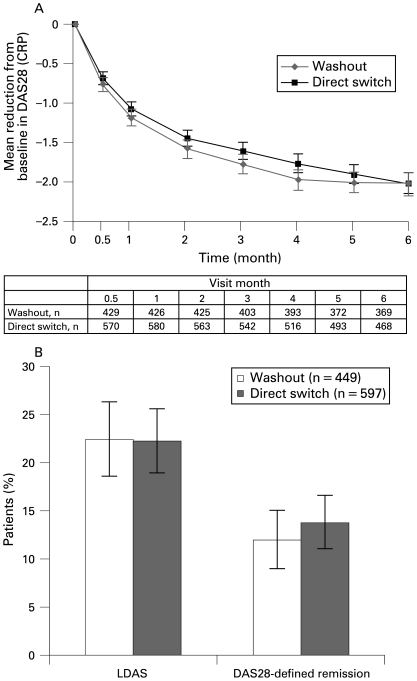
Efficacy following 6 months of abatacept treatment in washout and direct-switch patients. (A) Mean improvements in disease activity score in 28 joints (DAS28 (C-reactive protein; CRP)); (B) Percentage of patients achieving low disease activity state (LDAS; DAS28 (CRP) ⩽3.2) and DAS28-defined remission (DAS28 (CRP) <2.6). Error bars represent 95% CI.

At 6 months, the improvements in efficacy observed with abatacept were similar in washout and direct-switch patients (fig 2). The mean reduction (SE) in DAS28 from baseline was −2.0 (0.1) in both washout and direct-switch patients (fig 2A). Clinically meaningful improvements in DAS28 were reported in 56.1% of patients overall and in 59.5% and 53.6% of washout and direct-switch patients, respectively.

Overall, 22.4% of patients achieved LDAS and 13.0% achieved remission, including 22.5% and 12.0% of washout patients and 22.3% and 13.7% of direct-switch patients, respectively (fig 2B).

Post-hoc analyses of the mean change from baseline in DAS28 and the proportions of patients achieving LDAS and remission at 6 months were generally similar, regardless of the type of previous anti-TNF agent used (etanercept, infliximab or adalimumab) or the reason for the failure of previous anti-TNF therapy (ie, safety/tolerability versus efficacy). For the DAS28 mean change from baseline, LDAS or remission, the 95% CI did not overlap for patients who had failed one or three previous anti-TNF therapies (table 3).

**Table 3:** DAS28 responses at 6 months of abatacept treatment in patients stratified by number of previous anti-TNF agents, previous anti-TNF agent and reason for failure of previous anti-TNF agent

Patient subgroup	DAS28 response at 6 months (95% CI)		
	DAS28 mean change from baseline	LDAS % responders	DAS28-defined remission % responders
Type of previous anti-TNF*			
Etanercept (n = 278)	−2.0 (−2.2 to −1.8)	24.1 (19.1 to 29.1)	14.7 (10.6 to 18.9)
Infliximab (n = 348)	−2.1 (−2.3 to −2.0)	21.3 (17.0 to 25.6)	14.1 (10.4 to 17.7)
Adalimumab (n = 351)	−1.9 (−2.1 to −1.8)	23.4 (18.9 to 27.8)	11.1 (7.8 to 14.4)
No of previous anti-TNF†			
1 (n = 488)	−2.1 (−2.2 to −2.0)	24.8 (21.0 to 28.6)	15.8 (12.5 to 19.0)
2 (n = 340)	−2.1 (−2.3 to −1.9)	22.9 (18.5 to 27.4)	12.9 (9.4 to 16.5)
⩾2 (n = 540)	−2.0 (−2.1 to −1.8)	20.0 (16.6 to 23.4)	10.6 (8.0 to 13.1)
3 (n = 200)	−1.7 (−1.9 to −1.5)	15.0 (10.1 to 19.9)	6.5 (3.1 to 9.9)
Reason for failure‡			
Safety (n = 106)	−2.3 (−2.6 to −2.0)	30.2 (21.4 to 38.9)	16.0 (9.1 to 23.0)
Intolerability (n = 230)	−2.2 (−2.4 to −2.0)	22.6 (17.2 to 28.0)	13.5 (9.1 to 17.9)
Safety or intolerability (n = 305)	−2.2 (−2.4 to −2.0)	25.6 (20.7 to 30.5)	14.4 (10.5 to 18.4)
Efficacy (n = 952)	−2.0 (−2.1 to −1.9)	20.9 (18.3 to 23.5)	12.2 (10.1 to 14.3)

n Numbers are based on patients with available data (as-observed population). *Data were unavailable for 69 patients and are presented for the last anti-tumour necrosis factor (TNF) therapy received before initiating abatacept. †Data were unavailable for 18 patients. ‡Patients could have failed for more than one reason. DAS28, disease activity score in 28 joints; LDAS, low disease activity state.

#### HRQoL and physical function

**Figure 3:**
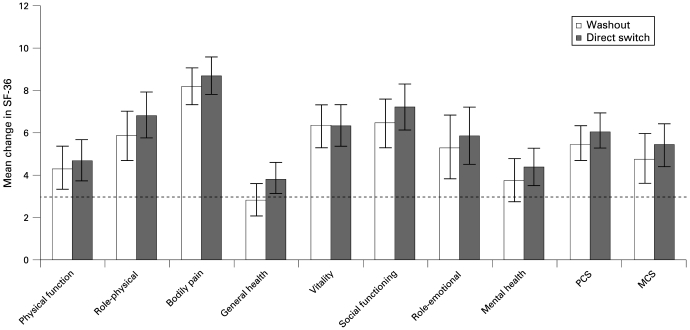
Effect of abatacept treatment on health-related quality of life (short-form 36 (SF-36) component scores and individual measures) in washout and direct-switch patients, following 6 months of abatacept treatment. Error bars represent 95% CI. A change in score of 3 or more was considered clinically meaningful (shown as dotted line). MCS, mental component summary; PCS, physical component summary.

Clinically meaningful improvements in physical function (HAQ-DI responses) were observed in 46.7% of the overall population and 46.3% and 47.1% of washout and direct-switch patients, respectively. Improvements in HRQoL are presented in fig 3.

### Monotherapy patients

#### Safety

Forty-three patients (20 washout and 23 direct-switch patients) received abatacept as monotherapy. AE and serious AE were reported in 83.7% and 9.3% patients overall, with 80.0% versus 87.0% and 5.0% versus 13.0% in washout versus direct-switch patients, respectively. One patient in the washout group discontinued because of an AE, no patients discontinued because of a serious AE. Serious infections were reported in one patient (gastroenteritis salmonella) from the direct-switch group only.

#### Efficacy

Efficacy in the patients receiving monotherapy was comparable to that seen in patients receiving background DMARD. For the overall, washout and direct-switch groups, respectively, mean improvements (SE) in DAS28 were 1.84 (0.19), 1.61 (0.25) and 2.05 (0.28); the percentages of patients with a clinically meaningful improvement in DAS28 (95% CI) were 48.8% (33.9 to 63.8), 50.0% (28.1 to 71.9) and 47.8% (27.4 to 68.2); mean changes (SE) in HAQ-DI were −0.38 (0.10), −0.29 (0.16) and −0.46 (0.13); mean changes (SE) in PCS were 4.80 (1.13), 3.65 (1.74) and 5.80 (1.47) and mean changes (SE) in MCS were 7.34 (1.93), 7.13 (2.38) and 7.53 (3.03).

## Discussion

The 6-month results of this study in abatacept-treated patients with RA and an inadequate response to anti-TNF therapy demonstrate acceptable safety and tolerability and clinically meaningful improvements in efficacy. Results were comparable in patients who had completed a washout of their anti-TNF therapy and in patients who switched directly to abatacept. These results confirm and extend previous findings from the ATTAIN study, which evaluated a similar patient population.^2^ Of the 391 patients in the ATTAIN study who responded inadequately to anti-TNF therapy, significantly more abatacept-treated patients achieved American College of Rheumatology (ACR)20 (primary endpoint), ACR50 and ACR70 responses compared with placebo (50.4%, 20.3% and 10.2% vs 19.5%, 3.8% and 1.5%, respectively).^2^ The ARRIVE study evaluated a larger population of 1046 patients, and included a substantially higher proportion of patients who had previously tried adalimumab compared with the ATTAIN study (51% vs 2%, respectively) as a result of the more widespread use of this anti-TNF agent at the time of enrollment. The ARRIVE study, unlike the ATTAIN trial, included patients who had failed anti-TNF therapy because of lack of efficacy, safety or tolerability reasons alone. Patients were also able to receive abatacept if they had a positive PPD test result, providing they had received one month or more of treatment for latent tuberculosis and had a negative chest *x* ray. Patients were not limited to particular background non-biological DMARD, and patients from the USA were permitted to receive abatacept as monotherapy, without any background DMARD. Patients included in the ARRIVE study exhibited levels of baseline RA disease activity closer to those commonly encountered in daily practice, with lower mean swollen and tender joint counts.^1^ ^2^ ^16^ Importantly, upon discontinuation of their anti-TNF treatment, patients were able to switch directly to abatacept on their next scheduled anti-TNF dose and were not required to undergo a washout.

In the ARRIVE study, the safety profile of abatacept was unaffected by a lack of washout period for anti-TNF agents before initiating abatacept. No increase in the overall frequency of infection was seen in patients switching directly to abatacept compared with those who completed a washout. Furthermore, incidences of infectious events in washout patients were comparable to those observed in direct-switch patients when assessed monthly after the initiation of abatacept therapy. There was a slightly higher frequency of infection in the first month of the study, regardless of whether or not patients completed a washout period. Despite the inclusion of patients testing positive for PPD, there were no cases of tuberculosis during the 6-month study. No opportunistic infections occurred. Four non-skin malignancies were reported in 1046 patients treated with abatacept: the use of large databases, such as RA registries, and also integrated analyses of existing trial data, will assist in the continued monitoring of rare events, such as malignancies. Autoimmune events occurred in less than 2% of patients, at a slightly higher frequency in direct-switch than washout patients. As direct switching to abatacept from anti-TNF therapy did not lead to an increase in AE, including infections, this study provides support for direct switching to abatacept in patients who have an inadequate response to anti-TNF therapy.

Consistent with previous findings in anti-TNF therapy inadequate responders,^2^ clinically meaningful improvements in disease activity, physical function and HRQoL were seen following abatacept treatment in the ARRIVE study. The efficacy of abatacept was similar in patients who had completed a washout of their anti-TNF therapy and in those who switched directly to abatacept. The efficacy of abatacept monotherapy was comparable to that observed in patients receiving background DMARD, although it should be noted that patient numbers were low in the former subgroup.

Abatacept provided considerable efficacy benefits irrespective of previous anti-TNF therapy experience, but the magnitude of improvement was greater in patients who had previously tried only one anti-TNF agent. Meaningful improvements in clinical measures of efficacy and HRQoL were observed in patients who had failed their previous anti-TNF therapy as a result of efficacy, tolerability or safety reasons; improvements were greater in patients who were safety failures. A previously published analysis of patients from the ATTAIN trial^17^ demonstrated that abatacept is efficacious regardless of whether patients did not respond to anti-TNF therapy or lost their response to anti-TNF therapy over time.

These data must be interpreted within the context of several limitations. The treatment period was limited to 6 months, potentially restricting the capacity to detect opportunistic infections and other infrequent AE. As the sample size for this study was not determined to detect differences between washout and direct-switch patients, the power for testing differences between these groups is unknown. However, as baseline demographics and disease characteristics were comparable, it is appropriate to compare the safety and efficacy between the two groups. Compared with results obtained from randomised clinical trials, the open-label nature of the trial design may introduce bias due to patient and/or investigator preconceptions. In addition, although this trial included patients with lower baseline disease activity than is often seen in randomised clinical trials, patients still had moderate-to-severe active RA, having failed one or more anti-TNF agent. These patients may represent a population with higher disease activity observed in daily clinical practice.

In conclusion, these results demonstrate the acceptable safety and tolerability and clinically meaningful efficacy benefits of abatacept in patients with an inadequate response to anti-TNF therapy, a population representative of those encountered in clinical practice. Moreover, these results support the clinical use of direct switching to abatacept from anti-TNF agents in patients who do not respond to, lose response to, or are unable to tolerate anti-TNF agents.
